# Sublingual methylcobalamin treatment in infants with prolonged jaundice due to vitamin B12 deficiency

**DOI:** 10.3389/fped.2026.1822723

**Published:** 2026-04-29

**Authors:** Sultan Aydin, Ayse Oz, Hayrullah Turkmen, Hicran Altin

**Affiliations:** 1 Department of Pediatric Hematology and Oncology, Antalya Training and Research Hospital, Antalya, Türkiye; 2 Department of Pediatrics, Antalya Training and Research Hospital, Antalya, Türkiye

**Keywords:** infant, jaundice, prolonged jaundice, sublingual methylcobalamin, vitamin B12 deficiency

## Abstract

**Objectives:**

Ineffective erythropoiesis resulting from vitamin B12 deficiency leads to hemolysis. The breakdown of erythrocytes increases plasma bilirubin levels, which can cause jaundice in newborns.

**Methods:**

This descriptive cross-sectional study was conducted at the Department of Pediatric Hematology, Antalya Training and Research Hospital. In total, 103 infants with prolonged neonatal jaundice due to vitamin B12 deficiency, defined as a serum level below 250 ng/L, were included. Participants were divided into two groups based on treatment type: sublingual methylcobalamin (Group 1, *n* = 72) and intramuscular cyanocobalamin (Group 2, *n* = 31).

**Results:**

The mean gestational ages were 38.36 ± 1.4 weeks in Group 1 and 38.87 ± 0.81 weeks in Group 2. The mean ages at diagnosis were 35.74 ± 13.17 days and 35.9 ± 11.73 days, respectively. The female-to-male ratios were 30:42 in Group 1 and 16:15 in Group 2. Mean serum vitamin B12 levels in Group 1 were 138.82 ± 50.09 ng/L before treatment, 656.86 ± 333.16 ng/L at 1.5 months, and 394.08 ± 169.94 ng/L at 3 months following sublingual methylcobalamin therapy. In Group 2, the mean levels were 165.71 ± 56.07 ng/L before treatment, 502.9 ± 196.07 ng/L at 1.5 months, and 345.77 ± 115.42 ng/L at 3 months after intramuscular cyanocobalamin administration.

**Conclusion:**

Vitamin B12 deficiency may be associated with prolonged jaundice in neonates. Early diagnosis of vitamin B12 deficiency enables timely initiation of sublingual methylcobalamin, a non-invasive and effective treatment option.

## Introduction

Prolonged jaundice is defined as a serum bilirubin level exceeding 5 mg/dL that persists beyond the 14th postnatal day in term infants or the 21st postnatal day in preterm infants. Hyperbilirubinemia lasting more than 14 days occurs in 15–40% of breastfed infants (1). Prolonged unconjugated hyperbilirubinemia may result from hemolytic conditions such as Rh or ABO incompatibility, glucose-6-phosphate dehydrogenase deficiency, congenital hypothyroidism, urinary tract infection, or inherited disorders such as Crigler–Najjar and Gilbert syndromes (2). Although breast milk jaundice is the most common cause of prolonged jaundice, its underlying mechanism remains unclear. It is a diagnosis of exclusion, established only after all other potential etiologies have been ruled out.

Vitamin B12 deficiency plays a significant role in various hematological and neurological disorders. It can cause mild hemolysis, leading to elevated bilirubin levels, particularly of the indirect (unconjugated) type. The underlying mechanisms are as follows: vitamin B12 deficiency impairs DNA synthesis, especially in rapidly dividing cells such as erythroid precursors; this results in ineffective erythropoiesis within the bone marrow, where many red blood cell precursors undergo premature destruction (intramedullary hemolysis); and the breakdown of these cells increases heme catabolism, thereby producing more unconjugated bilirubin. Consequently, erythrocyte lysis elevates plasma bilirubin levels, which may cause jaundice in newborns. Vitamin B12 deficiency is typically characterized by mild anemia, elevated LDH, low haptoglobin, increased indirect bilirubin, and the presence of hypersegmented neutrophils on peripheral smear. Based on these findings, vitamin B12 deficiency can be considered a potential cause of prolonged jaundice (3).

In a recent study measuring serum B12 and plasma homocysteine levels in both newborns and their mothers through newborn screening, 87 infants were confirmed to have vitamin B12 deficiency. The study demonstrated that identifying, treating, and monitoring marginal or low vitamin B12 levels in pregnant women can improve neonatal health outcomes and reduce healthcare costs. Cobalamin (vitamin B12) deficiency can be detected indirectly through newborn screening, although it is not a primary target of most programs. Screening often includes tests for methylmalonic acidemia and homocystinuria, both of which may result from inborn errors of metabolism or secondary vitamin B12 deficiency. Maternal vitamin B12 deficiency—particularly in vegan or malnourished mothers—is a common cause of secondary deficiency in newborns. Confirmatory testing involves measuring serum B12, MMA, and homocysteine levels in both the infant and mother. Although vitamin B12 deficiency is not a direct target of newborn screening, it can be incidentally identified through these metabolic markers. Early detection is crucial to prevent neurological damage. Several low- and middle-income countries are now considering expanded screening programs to facilitate earlier diagnosis and intervention (4).

Therefore, this study evaluated the efficacy of sublingual methylcobalamin treatment in infants with prolonged jaundice associated with vitamin B12 deficiency, a common nutritional deficiency in developing countries.

## Materials and methods

This retrospective, descriptive cross-sectional study was conducted in the Department of Pediatric Hematology at Antalya Training and Research Hospital between January 2020 and November 2022. Institutional review board approval was obtained from the Clinical Research Ethics Committee of SBÜ Antalya Training and Research Hospital (protocol no.: 2021–368; decision no.: 21/2; November 24, 2022). The study was conducted in accordance with the principles of the 1975 Declaration of Helsinki. Written informed consent was obtained from the parents or legal guardians of all participating infants.

*A priori* power analysis was performed using G*Power software (version 3.1.9.7) to determine the sample size required to achieve a statistical power of 0.80 with an alpha error probability of 0.05 and an assumed effect size of f = 0.25 for the primary interaction hypothesis. The analysis indicated that a minimum of 86 infants were required to achieve the desired statistical power. However, to enhance the generalizability of our findings, all eligible patients who received vitamin B12 treatment during the study period were retrospectively included. This comprehensive approach yielded a total study population of 103 infants treated at the Department of Pediatric Hematology for prolonged jaundice due to vitamin B12 deficiency, defined as a serum vitamin B12 level < 250 ng/L (5). Infants presenting with neurological symptoms who detected using Denver Developmental Screening Test due to of vitamin B12 deficiency or infants with other known causes of prolonged jaundice—such as hypothyroidism, urinary tract infection, hereditary hemolytic anemias (membranopathies, enzymopathies, etc.), or a positive direct antiglobulin test, Rh, ABO, or subgroup incompatibility were excluded from the study.

The study population was divided into two groups based on the treatment modality: a sublingual methylcobalamin group (Group 1, *n* = 72), and an intramuscular cyanocobalamin group (Group 2, *n* = 31).

**Sublingual methylcobalamin therapy group (Group 1):** Sublingual methylcobalamin was administered at a dose of 500 μg once daily for the first 1.5 months, followed by three times per week for the subsequent 1.5 months. Parents were instructed to avoid breastfeeding or giving the infants any fluids for 15 min after one spray was administered into the mouth.
**Intramuscular cyanocobalamin treatment group (Group 2):** Infants received intramuscular cyanocobalamin according to a structured regimen: 100 µg daily during the first week, every other day during the second week, twice weekly during the third week, once weekly during the fourth week, and once monthly for the subsequent 3 months.

Serum vitamin B12 levels were measured at baseline and after 1.5 and 3 months of treatment with either sublingual methylcobalamin or intramuscular cyanocobalamin. Demographic data, including maternal age and vitamin B12 level, and neonatal characteristics, such as gestational age, were collected. Laboratory parameters retrieved retrospectively from the hospital electronic database included hemoglobin (Hb), mean corpuscular volume (MCV), and red blood cell distribution width (RDW) from the complete blood count, as well as serum lactate dehydrogenase (LDH), total and direct bilirubin (Total Bil/Direct Bil), and serum vitamin B12 levels.

## Statistical analysis

Data analysis was performed using IBM SPSS version 23 (IBM Corp., Armonk, NY, USA). A significance level of *p* < 0.05 was applied. Normality of the data was assessed using the Kolmogorov–Smirnov and Shapiro–Wilk tests. Comparisons between two groups for non-normally distributed variables were conducted using the Mann–Whitney U test, whereas Friedman's test was used for repeated measures across three or more time points. Dunn's test was applied for multiple comparisons, and Yates’ correction was used for categorical variable comparisons between groups. Quantitative data are presented as mean ± standard deviation and median, with minimum and maximum values. Categorical data are reported as frequency (percentage).

## Results

Group 1 (sublingual methylcobalamin) included 72 infants (69.9%), whereas Group 2 (intramuscular cyanocobalamin) included 31 infants (30.1%) with prolonged jaundice due to vitamin B12 deficiency. The mean ages at diagnosis were 35.74 ± 13.17 days (range, 15–69 days) in Group 1 and 35.9 ± 11.73 days (range, 15–56 days) in Group 2. The female-to-male ratios were 30:42 in Group 1 and 16:15 in Group 2. The mean gestational ages were 38.36 ± 1.4 weeks in Group 1 and 38.87 ± 0.81 weeks in Group 2. There were no significant differences between the groups in most demographic or laboratory parameters, as summarized in Table 1. However, MCV and RDW were significantly higher in the sublingual methylcobalamin group (*p* < 0.001 and *p* = 0.004, respectively). Maternal serum vitamin B12 levels were lower in Group 1 (138.10 ± 44.33 ng/L; range, 59–248) compared with Group 2 (162.58 ± 49.56 ng/L; range, 80–280; *p* = 0.015).

**Table 1:** Demographic and laboratory characteristics of infants in the sublingual methylcobalamin and intramuscular cyanocobalamin groups.

Groups	Sublingual methylcobalamin group (Group 1)	Intramuscular cyanocobalamin group (Group 2)	*P*
	Mean ± SD	Mean ± SD	
Hb	11.88 ± 2.32	12.24 ± 2.18	0.115^a^
MCV	90.88 ± 5.71	77.68 ± 8.54	**<0.001** ^a^
RDW	14.64 ± 1.23	14.61 ± 3.06	**0**.**004**^a^
Total Bil	11.6 ± 1.6	11.13 ± 1.16	0.434^a^
Direct Bil	0.86 ± 0.22	0.84 ± 0.3	0.348^a^
LDH	288.21 ± 49.38	274.23 ± 51.32	0.052^a^
Reticulocyte (%)	2.03 ± 0.87	1.80 ± 0.24	0.051^a^
Age at diagnosis (days)	35.74 ± 13.17	35.9 ± 11.73	0.779^a^
Gestational week	38.36 ± 1.4	38.87 ± 0.81	0.076^a^
Maternal vitamin B12 level	138.10 ± 44.33	162.58 ± 49.56	**0**.**015**^a^

Hb, hemoglobin; MCV, mean corpuscular volume; RDW, red blood cell distribution width; Total Bil, total bilirubin; Direct Bil, direct bilirubin; LDH, lactate dehydrogenase; SD, standard deviation.

a Mann–Whitney U test.

The bold values is meaning of *P* value < 0.001.

In Group 1, mean serum vitamin B12 levels were 138.82 ± 50.09 ng/L before treatment, 656.86 ± 333.16 ng/L at 1.5 months, and 394.08 ± 169.94 ng/L at 3 months of sublingual methylcobalamin therapy. In Group 2, the corresponding values were 165.71 ± 56.07 ng/L before treatment, 502.9 ± 196.07 ng/L at 1.5 months, and 345.77 ± 115.42 ng/L at 3 months of intramuscular cyanocobalamin therapy (Table 2). The baseline serum vitamin B12 level was significantly lower in Group 1 compared with Group 2 (*p* = 0.018). After 1.5 months of treatment, Group 1 had a significantly higher mean serum vitamin B12 level than Group 2 (*p* = 0.046). No significant difference was observed between the groups at 3 months post-treatment (*p* = 0.221). Overall, serum vitamin B12 levels increased significantly in all patients across both treatment groups (Table 2).

**Table 2:** Comparison of serum vitamin B12 levels between sublingual methylcobalamin and intramuscular cyanocobalamin groups.

	Sublingual methylcobalamin group	Intramuscular cyanocobalamin group	*p*
	(Group 1)	(Group 2)	
Serum vitamin B12 level	Mean ± SD	Mean ± SD	
Baseline	138.82 ± 50.09	165.71 ± 56.07	**0** **.** **018** ^a^
1.5 months	656.86 ± 333.16	502.9 ± 196.07	**0** **.** **046** ^b^
3 months	394.08 ± 169.94	345.77 ± 115.42	0.221^b^
p	** < 0.001** ^c^	** < 0.001** ^c^	

a Independent two-sample t-test.

b Mann–Whitney U test.

c Friedman test.

The bold values is meaning of *P* value < 0.001.

## Discussion

Vitamin B12 is essential for DNA synthesis, a process required for cell division, through its role in the tetrahydrofolate pathway. Deficiency primarily affects tissues with rapid cell turnover, particularly the hematopoietic and gastrointestinal systems. Severe vitamin B12 deficiency can also disrupt the structure and function of cells in the nervous system (6). The severity and duration of deficiency influence the onset and prognosis of symptoms across multiple organ systems, with neurological findings primarily resulting from defective myelination. These deficiency-related manifestations often appear during the neonatal period (7). In infants, symptoms may include seizures, developmental delay, hypotonia, irritability, microcephaly, inability to hold the head, decreased eye contact, abnormal movements such as chorea or myoclonus, feeding difficulties, regurgitation, vomiting, weight loss, and chronic diarrhea. If left untreated, vitamin B12 deficiency can lead to cognitive impairment in subsequent months (8). Hematologically, deficiency causes megaloblastic anemia, presenting with pallor, weakness, and systemic findings such as hepatomegaly, cardiomegaly, and heart failure. Dermatological manifestations, including hyperpigmentation, glossitis, and stomatitis, have also been reported (9). Jaundice is another potential manifestation of vitamin B12 deficiency, typically associated with hemolysis. The underlying mechanisms involve ineffective erythropoiesis, intramedullary hemolysis, and bilirubin production leading to deficiency in B12 impairs DNA synthesis in erythroid precursors, the premature destruction of immature erythrocytes in the bone marrow and excessive release of biliverdin, which is subsequently converted to indirect bilirubin (10).

Neonatal jaundice occurs in approximately 60% of newborns, and in some cases, it progresses to pathological levels or becomes prolonged (2). Among infants with prolonged or pathological jaundice, the underlying cause remains unidentified in roughly half of the evaluations conducted to determine etiology (11). A deficiency of vitamin B12, which is essential for erythrocyte maturation and the DNA synthesis required for cell division and proliferation, can lead to excessive heme production due to red blood cell destruction, resulting in hyperbilirubinemia, particularly when combined with folic acid. Vitamin B12 also plays a critical role in efficient erythropoiesis. Deficiency of this vitamin is common across all age groups in many populations and can cause a mild elevation of indirect bilirubin due to hemolysis (12). Therefore, vitamin B12 deficiency may represent a silent but important cause of prolonged neonatal jaundice, and serum vitamin B12 levels may be assessed before attributing prolonged jaundice to breast milk jaundice.

Given its potential to cause serious hematological and neurological complications, early detection and treatment of vitamin B12 deficiency are essential. Intramuscular cyanocobalamin is widely reported to be the most effective treatment for childhood deficiency, typically administered daily during the first week, every other day in the second week, twice weekly in the third week, once weekly in the fourth week, and then monthly for a total of 6–12 months (13). However, repeated intramuscular injections are increasingly considered invasive, and complications such as sciatic nerve injury have prompted a search for more practical alternatives. Recently, sublingual methylcobalamin has gained attention as a non-invasive treatment option (14). Although no standardized pediatric dosing regimen exists, Orhan Kiliç et al. reported administering sublingual methylcobalamin to children aged 0–3 years at 1,000 µg once daily during the first week, every other day in the second week, twice weekly in the third week, and once weekly in the fourth week (15). In our study, sublingual methylcobalamin was administered at 500 μg once daily for the first 1.5 months, followed by three times weekly for the subsequent 1.5 months.

## Conclusion

In this study, we identified 103 infants with prolonged jaundice associated with vitamin B12 deficiency. Vitamin B12 deficiency serves as a potential cause that should be investigated when other common triggers of prolonged jaundice are ruled out. At the same time, our findings indicate that sublingual methylcobalamin is an effective and safe non-invasive treatment option for this condition, providing an alternative to invasive therapies such as intramuscular injections, which can cause muscle or nerve injury in infants. Additionally, our study highlights a practical, effective, and safe dosing regimen for sublingual methylcobalamin in infants, along with the precautions necessary to ensure its proper administration.

## Data Availability

The original contributions presented in the study are included in the article/Supplementary Material, further inquiries can be directed to the corresponding author.
